# Difficulties and Challenges of Anomaly Detection in Smart Cities: A Laboratory Analysis

**DOI:** 10.3390/s18103198

**Published:** 2018-09-21

**Authors:** Victor Garcia-Font, Carles Garrigues, Helena Rifà-Pous

**Affiliations:** 1 Departament of d’Enginyeria Informàtica i Matemàtiques, Universitat Rovira i Virgili (URV), 43003 Tarragona, Spain; 2 CYBERCAT-Center for Cybersecurity Research of Catalonia, 43003 Tarragona, Spain; 3 Internet Interdisciplinary Institute (IN3), Universitat Oberta de Catalunya (UOC), 08018 Barcelona, Spain; cgarrigueso@uoc.edu (C.G.); hrifa@uoc.edu (H.R.-P.)

**Keywords:** anomaly detection, information security, outlier detection, smart cities, support vector machines, isolation forest, wireless sensor networks, testbed

## Abstract

Smart cities work with large volumes of data from sensor networks and other sources. To prevent data from being compromised by attacks or errors, smart city IT administrators need to apply attack detection techniques to evaluate possible incidents as quickly as possible. Machine learning has proven to be effective in many fields and, in the context of wireless sensor networks (WSNs), it has proven adequate to detect attacks. However, a smart city poses a much more complex scenario than a WSN, and it has to be evaluated whether these techniques are equally valid and effective. In this work, we evaluate two machine learning algorithms (support vector machines (SVM) and isolation forests) to detect anomalies in a laboratory that reproduces a real smart city use case with heterogeneous devices, algorithms, protocols, and network configurations. The experience has allowed us to show that, although these techniques are of great value for smart cities, additional considerations must be taken into account to effectively detect attacks. Thus, through this empiric analysis, we point out broader challenges and difficulties of using machine learning in this context, both for the technical complexity of the systems, and for the technical difficulty of configuring and implementing them in such environments.

## 1. Introduction

In the last decade, major cities around the world have been installing technological elements in the streets in order to capture a plethora of urban information to populate smart city information systems that allow authorities to offer new and better services to citizens and to take a data driven city management approach. The installed elements belong to what is called the Internet of Things (IoT), wireless sensor networks (WSNs) being one of the most extended technologies in this area. In smart cities, popular proposals use WSNs to improve parking systems [1] or public lighting [2].

In order to install these networks in the streets, many public administrations have outsourced the installation and operation of these services to external providers. This has raised, however, concerns related to the quality of data [3]. Outsourcing WSN operation usually results in the loss of control over network devices and this, in turn, causes a lack of visibility over the potential data-quality problems affecting these devices. Even when external providers implement proper security mechanisms to ensure data integrity, in practice, smart city administrators cannot determine the extent to which received data are precise and accurate. In fact, the Royal Academy of Engineering has identified data quality as one of the six major barriers to effectively optimize smart infrastructures [4].

To counter this problem, smart city IT administrators need to apply anomaly detection measures, so that they can detect when data stops following the expected patterns. The role of the data analyst, or data scientist, is here of utmost importance. The huge amount of data generated by the smart city WSNs have to be examined to determine the extent to which they are reliable and can be trusted.

An overly simplistic approach to this problem could lead to the definition of thresholds on key system variables to detect abnormal behaviours. However, Ref. [5] has shown that complex environments with large volumes of heterogeneous data require more powerful algorithms. The anomaly detection mechanisms must support a multivariate analysis where data patterns are dynamic and where no simple rules can be used to determine whether the behaviour is normal or when it is deviating from the standard. By using these algorithms, the normal state of the system can be learned periodically. This is performed through training mechanisms, which are used to create mathematical models that represent the normal state. Then, these models can be used to determine when a new value must be considered an outlier.

In these scenarios, machine learning-based techniques can undoubtedly give very good results, considering the success that machine learning has seen in many other areas of computer science, such as product recommendations systems [6,7], optical character recognition systems (e.g., [8,9]), natural language translation [10], and also spam detection [11]. Certainly, a smart city is a complex environment where we can find a huge volume of heterogeneous data. Additionally, most equipment belonging to the smart city’s WSNs is especially vulnerable to attacks due to its easy access in public spaces. Thus, the use of advanced anomaly detection techniques in smart cities is critical to contributing to their data reliability and trustworthiness.

The main purpose of this article is to evaluate the use of machine learning techniques for anomaly detection in this context. In the WSN research field, machine learning has already been used successfully in the past to detect attacks against the networks and it is very often used to implement intrusion detection systems (IDS) [12,13,14]. However, these IDS are designed for controlled environments, where WSN administrators have full access to their nodes. In this article, we want to analyze the use of machine learning in smart city environments, which are much more complex in terms of heterogeneity, number of nodes, accessibility to providers’ infrastructure, etc.

To do so, firstly, we have used a simulation environment in a laboratory with real equipment. This environment has allowed us to simulate attacks to the network without compromising the normal operation of a real smart city. We have then used the data received from the sensors to compare and evaluate two widely known machine learning techniques (one-class support vector machines (OC-SVM) and isolation forests). This analysis has shown that it is possible indeed to use machine learning to implement anomaly detection systems for complex environments such as smart cities.

Secondly, our work has shown that the use of these algorithms in this type of scenarios is complicated, and several things must be taken into account to avoid drawing wrong conclusions. Applying machine learning techniques is not as simple as calling a method of a specialised library. The complexity lies not only in the need to understand how to use a certain machine learning algorithm, but also in taking into account the difficulties associated with their application in a smart city context.

In this paper, we present the difficulties posed by the use of machine learning in these scenarios and the challenges that lie ahead: the availability of anomaly-free data, the use of the right machine learning technique for each situation, the aggregation of data in appropriate time windows, among others.

The rest of the paper is structured as follows: Section 2 contains background and related work; Section 3 describes our laboratory simulation environment; Section 4 shows the attacks performed in the laboratory; Section 5 provides details of the proposed anomaly analysis to detect the attacks. In Section 6, we evaluate the experiment results, discuss the testbed experience and we highlight main challenges in this context. Finally, Section 7 concludes the paper.

## 2. Background

Environmental sustainability, economic growth, technological progress and population growth are, among many others, new challenges that contemporary cities need to face [15]. In order to address these challenges, many projects propose to use technological elements to gather city data and interact with citizens and urban institutions and, in this way, create new services and explore efficient ways to improve existing ones. These projects are normally included in what is popularly known as smart city. Some popular smart city proposals are: the PlanIT Urban Operating System [16], Rio Operation Center [17], Ubiquitous city (u-city) [18] or SmartSantander [19,20].

In order to gather street data, it is common that WSNs and other IoT elements are deployed in the urban landscape. These elements are generally installed and operated by city councils and several external providers. Generally, different providers use different technologies, which have to not only coexist in the same physical space, but also they have to share certain equipment (e.g., gateways, electrical grid) in order to function properly and deliver collected data to the smart city data center. From the data analyst perspective, this creates a scenario difficult to analyse: data are dynamic, heterogeneous, and can easily be compromised.

Regarding security, the plethora of different technological solutions deployed in a smart city makes it impossible to install a reduced set of security countermeasures. Furthermore, security problems are especially important in WSNs. These networks are made of low-power devices that rely, in many cases, on multi-hop capabilities to build an extensive network and deliver packets from the most remote nodes to a base station. In addition, nodes are frequently battery-powered and, therefore, are designed also with restringed processing capacity to save power. The limited computational and energetic constraints of nodes are an obstacle to applying conventional computer network security countermeasures in WSNs. Additionally, in these networks, nodes become more vulnerable when they are placed in unprotected environments like streets. In these circumstances, attackers can easily capture nodes, access confidential information in their memory (e.g., cryptographic keys) and reprogram their behavior. Attackers may also benefit from the wireless nature of the communications to intercept the messages or to obstruct frequency bands to impede the proper reception of some packets. In [21,22], the authors survey the most popular attacks on WSNs.

Given the above, the task of data analyst or IT administrator is to ensure that the data collected in the server is trustworthy and reliable. This means, on the one hand, that external providers must apply proper security mechanisms to their networks, but it also means that sound anomaly detection mechanisms must be put in place. However, this anomaly analysis is far from simple and requires sophisticated techniques, as we show below.

A lot of research has been carried out to tackle the anomaly detection problem in wireless sensor networks. In [23], the authors survey the different techniques and propose a categorization based on the model learned by each algorithm. On the one hand, statistical techniques use a density distribution representing the behaviour of the data. Once the parameters of the distribution are estimated, anomalies can be detected as data points with low likelihood.

On the other hand, non-parametric techniques do not rely on the availability of parameters describing the data behaviour. Some of the best known non-parametric techniques are rule-based approaches, data clustering or machine learning. Rule-based approaches use signatures (or attack patterns) of previously known attacks to identify anomalies. In data clustering, data are grouped into clusters according to some measurement. Data points encountered outside these clusters are labeled as outliers. Finally, machine learning techniques generate a data model that can be used to identify outliers. This model can be created from labeled data (data tagged as normal or abnormal) when supervised learning is used. Conversely, when using unsupervised learning, the model can be generated without prior knowledge of the analysed data.

The problem of statistical approaches is that data distributions are usually unknown because sensors are mobile or the networks are dynamic. Rule-based approaches have a similar drawback: predefined signatures are of no use against unknown attacks or network changes. By using clustering or machine learning techniques, on the other hand, data can be modelled even when its distribution or behaviour is unknown, and models can be updated periodically to adapt to new data patterns. This is the reason why these type of techniques are usually preferred in complex heterogeneous environments that evolve over time.

The problem of data analysis from complex environments has also been studied taking into account their big data nature. In [5], Suthaharan reviews different machine learning techniques and their applicability in scenarios with high amounts of data. The author highlights the excellent classification accuracy of support vector machines (SVM)-based approaches, but also warns about the computational complexity of these algorithms in a big data context, since these techniques have a high computational cost. In [24], Hayes et al. propose a system based on two detection levels that are used to support maximum scalability for large amounts of data. The first level is based on using a historical data model that provides very fast detection rates. The second level, which is more computationally expensive, uses k-means clustering to sharpen previous detection results using contextual information such as location, time of the day, etc.

Smart cities are, by definition, complex environments where one can find high volumes of data with a dynamic and heterogeneous nature. The current state of the art reviewed above clearly shows that data analysis and anomaly detection cannot be based on statistical or rule-based approaches alone. These approaches should be combined with sophisticated techniques based on machine learning, as suggested in [25,26].

As we previously introduced, the main goal of this paper is to demonstrate to what extent this is really feasible in a smart city context, not theoretically but in practice. In order to do so, we have carried out an anomaly analysis testing two well-known machine learning algorithms, SVM and isolation forests, which are described in the next section.

In order to conduct our test, we might have used deep learning, a subset of machine learning which uses a hierarchical level of artificial neural networks to carry out the data analysis. In this regard, recent advances show that this is a very promising field. As an example, algorithms based on deep belief networks [27] convolutional neural networks [28] or recursive neural networks [29] have been used successfully in several scenarios to improve the performance obtained with previous machine learning techniques. However, the use of deep learning for anomaly detection is a research field still in its infancy. Deep learning techniques, in general, require costly training processes, which is something easily attainable in fields such as computer vision, speech recognition, etc. However, in the case of smart city WSNs, obtaining large training datasets is much more complex due to their heterogeneous nature, which makes every smart city environment different from the rest. Therefore, the use of deep learning techniques has been considered inappropriate for the purpose of our work.

### 2.1. One-Class Support Vector Machines (OC-SVM)

Basically, classification techniques based on machine learning require two steps. First, a dataset is used to train a learning model. Then, the trained model is used to classify new data samples. Several features define each sample of the datasets. Classification techniques based on support vector machines (SVM) have proven to be effective in several contexts related to intrusion detection [12,30]. The SVM classification process represents the training dataset in a *n*-dimensional vector space, *n* being the number of features of the training data. Then, it defines a hyperplane (i.e., a *n-1* dimensional plane) that separates (with a maximum margin) the samples from the different classes. The support vectors are the subset of training samples that are near the hyperplane and that define it. Finally, the hyperplane acts as a frontier to classify other samples.

In our work, we have used one-class support vector machines (OC-SVM), which are a special case of semi-supervised SVM that do not require labeled data. OC-SVM build a frontier to classify new samples as normal or outlier. In SVM, different types of kernel functions are available to build the most adequate hyperplane for each application. In this work, we have used a radial basis function (RBF) kernel, which can learn complex regions [31]. In order to build the frontier, RBF kernel OC-SVM use basically two parameters [32]: ν and γ. On the one hand, ν defines the maximum fraction of outliers present in the training data. On the other hand, γ establishes the influence area of the support vectors on the classification. Generally, increasing the value of γ implies adjusting the frontier closer to the training samples. This reduces the number of misclassified outliers as normal samples. However, increasing γ too much causes the training data to be overfitted. A usual approach to select optimum parameters is grid search [33]. This method uses a grid with parameter values that is exhaustively explored in order to select the values that give the best performance of the SVM over a set of samples.

### 2.2. Isolation Forest

Isolation forest [34] is an unsupervised outlier detection technique that, unlike OC-SVM, does not construct a model based on the normal behaviour of a dataset. Instead, this technique builds a model that explicitly isolates anomalies. This has the advantage that models are already optimized to find anomalies. Moreover, compared to other popular anomaly detection techniques, such as ORCA, local outlier factor (LOF) and random forest, a key advantage of isolation forest is not only that it performs better, but also that requires a shorter processing time. In addition, unlike higher computationally complex methods, an isolation forest is capable of handling high dimensional spaces and large data sets.

Isolation forest finds anomalies building an ensemble of iTrees for a data set. Building an iTree consists of recursively building a tree structure, where in each step a feature is randomly chosen and also a splitting value between the maximum and the minimum of that feature is randomly selected. In this way, for each iTree in the ensemble, it is possible to compute the path length from the root to a leaf that is required to isolate each sample of a data set. A short average path length in the ensemble means that it is easy to isolate that sample and, therefore, it points out an anomaly. This technique requires two parameters: the number of trees in the ensemble and sub-sampling size to build each tree. In our work, we have set the parameter values to the values recommended by the authors proposing isolation forest in [34] (i.e., ensemble size of 100 trees and minimum sub-sampling size of 256 samples).

### 2.3. Smart City Testbeds

The growing awareness that data reliability and security are of utmost importance has motivated the development of several initiatives focussed on testing new protocols, devices, security measures, etc. in smart city contexts. Basically, these initiatives can be divided into three types: simulations (1), urban testbeds (2) and laboratory testbeds (3). Each environment has some advantages if used at the right time of project maturity [35,36].

Initiatives based on simulations (1) usually have the main advantage of flexibility. Teams in charge of simulations can easily change test settings and also change the configuration of the protocols or the devices included in the test scenario, add or remove nodes, change their geographic location, etc. There are powerful simulators for the IoT, such as NS-2 [37], Cooja [38], OMNET++ [39] or Castalia [40]. However, the lack of realism is the main drawback of this type of initiatives. This type of simulation is appropriate to test algorithm and protocols; nonetheless, they are not enough to fully validate proposed solutions in complex environments.

On the other hand, urban testbeds (2) aim at deploying a large amount of IoT devices in one or several cities. For instance, City of Things [41] in Antwerpen, Belgium, and SmartSantander [20] in Santander, Spain, are two projects where a broad range of technologies have been deployed to create large smart cities for research purposes. Contrary to what happened with (1), this type of testbed has the advantage of realism. Nonetheless, these initiatives generally lack flexibility to adapt the tests to other configurations or equipment beyond those deployed since the beginning. Moreover, setting up and running the experiments tend to be more expensive and requires more time in real environments than in simulators.

Halfway between (1) and (2) lay the initiatives deploying testbeds in laboratories (3). Although this type of testbed is limited in terms of scalability and interaction with the citizens, it has the advantage of using real hardware in a controlled environment. In addition, laboratory testbeds are more flexible than urban testbed, since it is much easier to add/remove devices, deploy new protocols, etc. In addition, testing in a laboratory allows experimenting with extreme conditions that may affect other services beyond those directly involved in the testbed. For example, jamming attacks not only interfere with the communication of testbed nodes, but also with other smart city devices communicating in the same frequency band, and it could even disrupt the neighbors’ Wi-Fi connections.

Regarding laboratory testbeds, it can be seen that in many cases, the test scenarios are designed to test a single smart city use case, such as the experiment in power systems in the SmartGridLab [42] project. Testbeds of this type provide valuable data for improving a particular service, device or protocol, but they take a silo approach.

On the other hand, other laboratory testbeds take a more holistic approach. These initiatives consider the heterogeneity of the smart city and seek to test technological elements taking into account their interaction and integration with other devices. An outstanding initiative of this type is I3ASensorBed [43]. This is a testbed focused on testing body area networks (BANs) and WSN communications. Another initiative with a stronger focus in cybersecurity is the Smart City Testbed Laboratory [44] deployed by the Center for Cyber Security at New York University Abu Dhabi (CCS-AD). With the aim of improving the safety of cyber-physical systems from a multidisciplinary approach, this laboratory incorporates typical hardware from different domains of a smart city, interconnecting the devices and exchanging data. In order to gather data and to monitor the devices, the laboratory includes an IoT element control platform.

In order to test the feasibility of the aforementioned machine learning algorithms, in this article, we take a laboratory testbed approach very similar to the Smart City Testbed Laboratory. In addition, as shown later on, in order to mitigate the lack of interaction with the city dynamics, our tests have been carried out using values captured from real smart city services, thus replicating real transmission patterns.

## 3. Simulation Environment

To design the simulation environment created to perform our tests, we have replicated in the lab a standard area of a smart city. To this end, we have emulated certain characteristics that make the smart city a very complex scenario, especially regarding WSN security. The main features of this simulation environment are:

- It is highly heterogeneous, which means it includes:
  - Different protocols (i.e., ZigBee, 6LoWPAN, WiFi).
  - Different hardware (i.e., Z1, Waspmotes).
  - Different sensor configurations (i.e., battery or grid powered, nodes with different computational limits, low/high transmit power).
  - Different network topologies (i.e., star, mesh).
  - Different routing path to the smart city servers (i.e., direct routing to the city servers, indirect routing through a third party server).
  - Different types of sensors (i.e., electrical measurements, environmental).
- It has different components physically sharing an area. Regarding security, this is relevant because an attack affecting an area can affect several nodes from different networks.
- It has different WSNs sharing the radioelectric space. Here, an attack affecting a particular frequency band can disrupt communication in several networks.
- It has different WSNs sharing a single gateway. An attack aiming at the gateway can cause packet loss of distant nodes from different networks.

**Figure 1:**
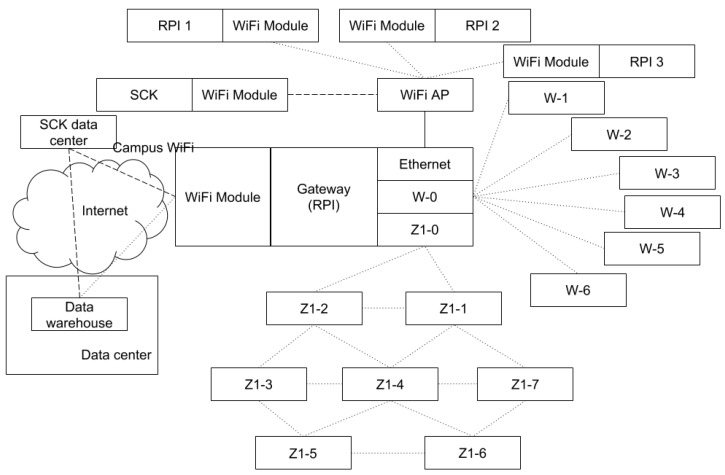
Technical schema of the testbed.

Figure 1 shows a technical representation of the simulation environment and Table 1 describes in more detail the most relevant characteristics of the included networks. As the figure shows, the WSNs send data (i.e., sensor readings and network status data) from the sensors to the gateway. The gateway, in turn, re-sends the data through the Internet to a server in the lab, where the data are stored in a data warehouse. On a large scale, this server would be the equivalent to the central servers of the smart city. It is worth noting that the sensor networks are configured either in a star topology or in a mesh topology, which are the most common topologies in smart cities. The networks configured in a star topology use, on the one hand, a point-to-point TCP socket connection over WiFi between each sensor node and the gateway in the RPI network. On the other hand, in the Libelium network, the ZigBee protocol coordinates an adequate transmission between all the nodes and the gateway [45]. In both cases, the gateway network address has been manually configured in the sensor nodes. The network configured in a mesh topology, i.e., the Zolertia network, uses 6LoWPAN to enable multihop capabilities, implementing UDP in the transport layer and IPv6 in the network layer with RPL (Routing Protocol for Low power and Lossy Networks) [46]. In order to force sensors nodes to communicate using multiple hops in a reduced scenario, such as the laboratory, the transmission power of the nodes in this network has been set particularly low.

In order to add more realism to the executed simulations, the sending patterns of the nodes and the content of the sensor readings have been set up cloning the behavior of real sensors from the smart city of Barcelona (excluding the SCK, which reads actual environmental data from the lab). Nodes have also been configured to send network status data gathered by the sensors. Moreover, in order to collect a larger volume of data, simulation speed has been triplicated. This means that one week of simulation data corresponds to three weeks of actual city data.

## 4. Attack Description

**Figure 2:**
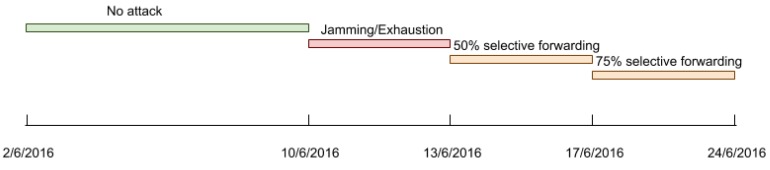
Attack execution cronogram.

Once the simulation environment has been set up, we have implemented some attacks so that we can later apply anomaly detection techniques and analyse their results. Specifically, we have implemented three denial of service (DoS) attacks against the aforementioned networks. The execution of these attacks has been divided in different stages that are depicted in Figure 2. The first stage was an attack-free execution and the attacks were executed from the second to the fourth stage.

The following sections provide more details about the implemented attacks.

### 4.1. Stage 2: Jamming

**Figure 3:**
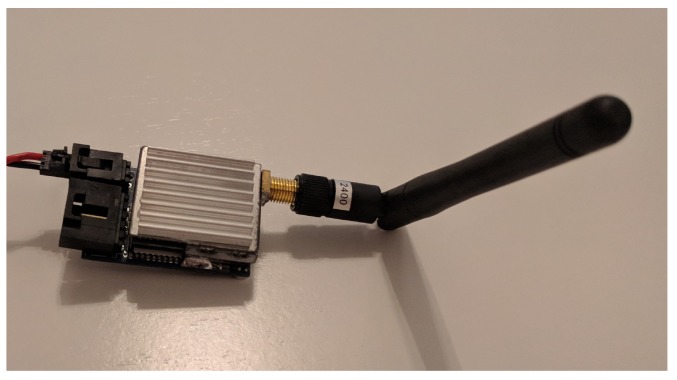
Boscam-TS321 2.4 GHz video transmitter.

In a random jamming attack, attackers broadcast a high-power signal in a random manner in order to create interference with current transmissions. In our case, we have implemented the jamming attack using a Boscam-TS321 [50] (Figure 3). This device is a video transmitter with a 500 mW power output. The transmitter uses radio frequencies ranging from 2414 MHz to 2510 MHz divided into eight different channels. From this moment on, we will refer to this transmitter as jammer.

**Figure 4:**
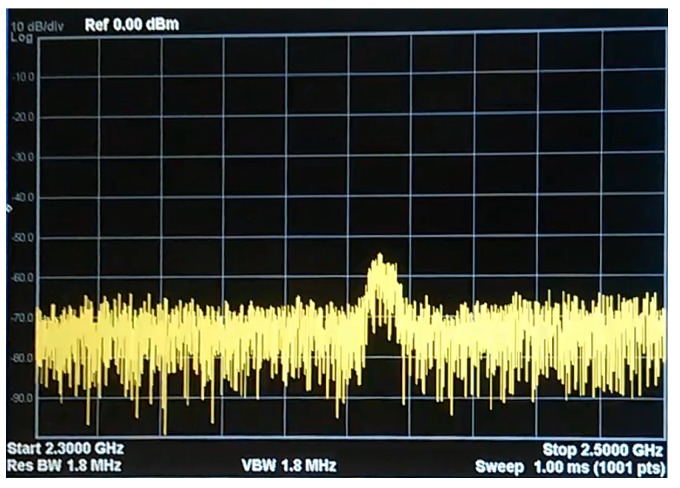
Radio spectrum between 2.3 GHz and 2.5 GHz at the transmission time of a Waspmote.

**Figure 5:**
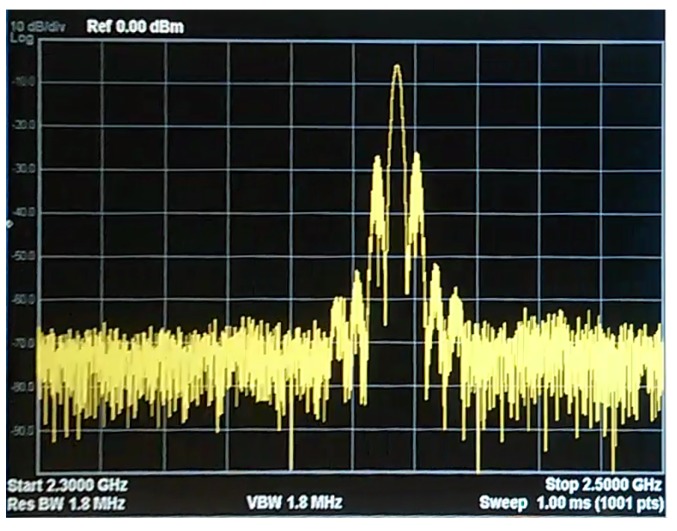
Radio spectrum between 2.3 GHz and 2.5 GHz with the jammer on.

As Figure 4 and Figure 5 show, the jammer transmits a high-power signal that is able to disrupt and cover up any other signal from the nodes in the simulation. In order to achieve this, the same radio frequency must be set up in the jammer and in the sensor nodes. However, some WSNs implement frequency hopping spread spectrum [51], a security protocol that allows nodes to agree on changing the transmission channel to avoid certain types of jamming attacks. In any case, using three Boscam-TS321 transmitters would be enough to block the entire spectrum and avoid the sensor motes in the 2.4 GHz band to find a non occupied channel. In our simulation, to avoid blocking our campus WiFi, we have used only one jammer, and we have disabled the frequency hopping feature of our nodes. Since all the nodes in the simulation are configured to work in the 2.4 GHz band, this attack affects all the networks of our scenario.

### 4.2. Stages 2 and 3: Selective Forwarding

In a selective forwarding attack, a compromised gateway is used to discard part of the packets that it should forward to the next hop. To do so, attackers must have had access previously to one or more nodes with forwarding capabilities. It should be noted that this is possible in the context of a smart city, where nodes are often placed unprotected in the streets. Then, once attackers have taken control of a gateway, they can reprogram it to discard some received packets instead of forwarding them. In our case, as shown in Figure 2, we have implemented two different selective forwarding attacks in the third and fourth stage. The attacks have targeted the Z1 network compromising its gateway. In the first attack, the gateway randomly discards 50% of the received packets coming from any other Z1 node. In the second stage, the gateway randomly discards 75% of the packets.

In order to implement this attack in the Z1 gateway interface, we have modified the *packet_input* function in the Contiki (Contiki is the Z1 nodes’ operating system) file “/core/net/ip/tcpip.c” (tcpip.c file contains the implementation of the TCP/IP protocol), as it can be seen in Listing 1.

Listing 1: Modification of the *packet_input* function in the tcpip.c Contiki file to perform a 50% selective forwarding attack.
static void
packet_input(void)
{
//Modified section to perform a selective forwarding attack
unsigned short threshold_rand_max;
unsigned short random_number;
random_number = random_rand();
//Next line controls the percentage of discarded packets (50% in this case)
threshold_rand_max = RANDOM_RAND_MAX*0.5;
if (random_number<threshold_rand_max){
//The node performs the attack and does not forward a packet
   uip_len = 0;
}
//End of the modified section to perform a selective forwarding attack
 
#if UIP_CONF_IP_FORWARD
  if(uip_len > 0) {
[...]
#endif /* UIP_CONF_IP_FORWARD */
}

## 5. Anomaly Detection

In this section, we will explain how the data generated in the four previously mentioned stages have been processed. Firstly, we have carried out a visual analysis, as shown in Section 5.1. Then, Section 5.2 describes how we have used two different machine learning techniques to compare their results and draw conclusions on the feasibility of their use in a smart city scenario.

### 5.1. Visual Analysis

As a first anomaly detection technique, before proceeding to the machine learning analysis, we have aggregated data in different time intervals in order to perform a preliminary visual analysis.

**Figure 6:**
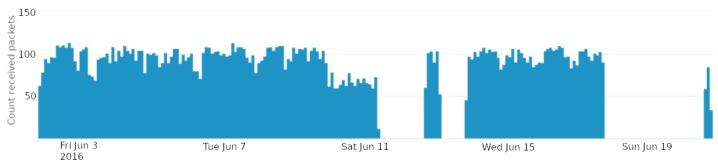
Number of packets received from the SCK node aggregated every two hours.

**Figure 7:**
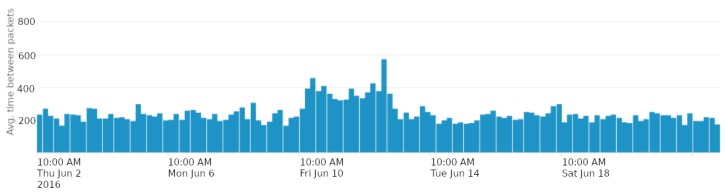
Average time between consecutive packets in the RPI network. These data have been aggregated every 4 h.

Figure 6 plots the number of packets received by the SCK node every two hours. Below, Figure 7 plots the average time between consecutive packets in the RPI network. In this second case, data have been aggregated in 4 h intervals. These figures show the effects that the attacks have had on two WSNs. For example, in the first plot, we can easily identify three moments in which the SCK node has stopped transmitting. The first two times, the data warehouse has stopped receiving SCK data due to the unavailability of the third party internet service from which data are collected. The third time has been due to the saturation of the SCK device.

A bit more subtle analysis of the plots shows the effect of the jamming attack. In Figure 7, around 10 to 13 June, the time between packets increases. Around the same time, in Figure 6, the number of received packets is greatly reduced.

This type of visual analysis is useful for smart city IT administrators or data analysts to quickly review the state of their systems. Furthermore, analysts should define certain rules to automatically get alerted in case of a single or a correlation of variables exceeding certain thresholds. However, network data can be very dynamic and their behaviour can evolve over time. Thus, defining thresholds is often too complex or unfeasible in scenarios such as the smart city. Moreover, security attacks can have unexpected effects without a clear detrimental impact on a single variable, creating instead an anomalous situation that can only be detected if several variables are analyzed at the same time. Therefore, as we previously mentioned, the use of more advanced techniques is very important. In the next section, we use machine learning techniques to perform an anomaly detection analysis in the simulation environment we have presented above.

### 5.2. Attack Detection with Machine Learning

In this section, we describe how we have used OC-SVM and isolation forest algorithms to conduct our anomaly analysis. Moreover, as shown later on, we will see how different approaches have been used to divide the WSN data at the time of computing the models. The utility of these models regarding the anomaly analysis will be discussed in Section 6.

The scripts implemented to run the algorithms have been written in Python 3.6, and the main library used for the machine learning computations is scikit-learn 0.19.0 [32]. The code and data obtained from the tests are accessible at [52].

In the following sections, we will describe the analysed datasets and a summary of the three steps taken to prepare and evaluate the machine learning models: training, validation and testing.

#### 5.2.1. Dataset Description

Before training the machine learning models, the data gathered from the WSNs have been aggregated by network in 30 min intervals. Other time intervals are also possible, but we have considered 30 min is a reasonable trade-off between setting shorter intervals, which overly reduce the variability of the resulting data, and longer time intervals that reduce the sampling set. Table 2 shows a summary of the new variables extracted from each network. As shown in the table, the values of the sensor readings have been ignored, since the implemented attacks have a denial-of-service effect and, therefore, the values recorded by the sensors have not been altered.

**Figure 8:**
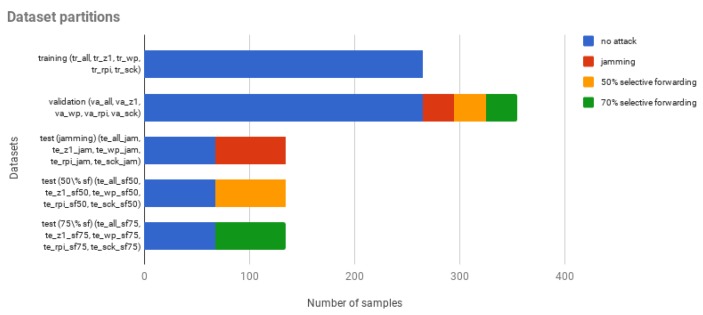
Dataset partition.

Afterwards, the data obtained from the previous aggregation process have been divided into several dataset partitions, as Figure 8 shows. These partitions have been used to analyse our data using two different approaches: (1) all variables from all the networks together are used to generate a single model; (2) the variables from the different networks are used separately to generate different models.

For the first approach, we have created five datasets: a training dataset (*tr_all*), a validation dataset (*va_all*) and a test dataset for each type of attack (*te_all_jam*, *te_all_sf50*, *te_all_sf75*). On the other hand, for the second approach, we have created 20 datasets. A training dataset for each of the four networks (*tr_z1*, *tr_wp*, *tr_rpi*, *tr_sck*), a validation dataset for each of the four networks too (*va_z1*, *va_wp*, *va_rpi*, *va_sck*) and a test dataset for each of the three attacks and each network (*te_z1_jam*, *te_wp_jam*, *te_rpi_jam*, *te_sck_jam*, *te_z1_sf50*, *te_wp_sf50*, *te_rpi_sf50*, *te_sck_sf50*, *te_z1_sf75*, *te_wp_sf75*, *te_rpi_sf75*, *te_sck_sf75*).

#### 5.2.2. Training Phase

The two techniques, OC-SVM and isolation forest are machine learning algorithms that can be trained with labeled data from just a single class: the attack-free period in our case (Figure 2). This is necessary in our scenario because labelling data from every possible attack or network malfunction is not possible, especially in the case of unknown attacks or abnormal network behaviours. Thus, with these techniques, we have generated models that are able to predict whether new data belong to the same class as the training data. As the training data are attack-free, any data that does not belong to this class can be considered an anomaly. Obviously, these models must be retrained periodically, since smart city WSNs evolve over time.

#### 5.2.3. Validation Phase

The validation datasets have been used basically to find appropriate values for the OC-SVM parameters (i.e., ν, γ). In order to tune these parameters, we have executed a grid search. Firstly, we have built a grid with many possible values for the parameters. Then, we have exhaustively explored the grid, creating new OC-SVM models to find the best combination of parameters. In each iteration of this algorithm, we have evaluated a pair of values using cross validation: one for the parameter ν and one for the γ.

For the cross validation, we have divided the attack-free samples of the validation dataset in 10 parts. Then, we have built a new training dataset with nine of these parts, and the remaining part has been combined with random attack samples from the validation dataset. With the resulting data, we have trained a new model, evaluating its performance with the f1-score metric (next section contains more details about metrics). This process has been iterated 10 times, thus obtaining different models trained with the same parameter values using different parts of the validation dataset. Finally, the f1-score results of the 10 models have been averaged.

As we can see, this grid search with cross validation has allowed us to select a good combination of ν and γ parameters (the one with the best f1-score average), and these are the parameters that have been used in the next test phase.

#### 5.2.4. Test Phase

In this section, we will show the final results of our study, which have been obtained using the test datasets. As we can see, different samples have been used for the training (i.e., training datasets), different samples to select the parameters of the algorithms (i.e., validation datasets), and different samples to evaluate the models (i.e., test datasets). This reduces the probability of overfitting. Additionally, as Figure 8 shows, the test datasets contain the same proportion of attack and attack-free samples. In this way, we have avoided having skewed datasets that could lead to biased conclusions.

To evaluate the predictive capabilities of the models, we have used the metrics summarized in Table 3, which have been widely used to assess anomaly detection techniques [53]. Each prediction of the model has been classified as a false positive (FP), if the prediction is ‘anomalous’ and the sample comes from the attack-free period; a true positive (TP), if the prediction is ‘anomalous’ and the sample comes from an attack period; a false negative (FN), if the prediction is ‘normal’ and the sample comes from an attack period; and a true negative (TN), if the prediction is ‘normal’ and the sample comes from the attack-free period.

**Figure 9:**
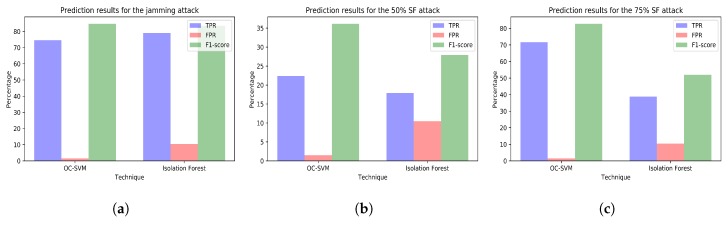
(**a**) prediction results for the jamming attack period using the variables from all the wireless sensor networks (WSNs) (training with dataset *tr_all* and test with dataset *te_all_jam*); (**b**) prediction results for the 50% selective forwarding attack period using the variables from all the WSNs (training with dataset *tr_all* and test with dataset *te_all_sf50*); (**c**) prediction results for the 75% selective forwarding attack period using the variables from all the WSNs (training with dataset *tr_all* and test with dataset *te_all_sf75*).

Below, we present the prediction results obtained in our experiments. The next section discusses these results. Figure 9a–c plot the prediction results for the three attacks comparing the two techniques using together all the variables from all the WSNs.

**Figure 10:**
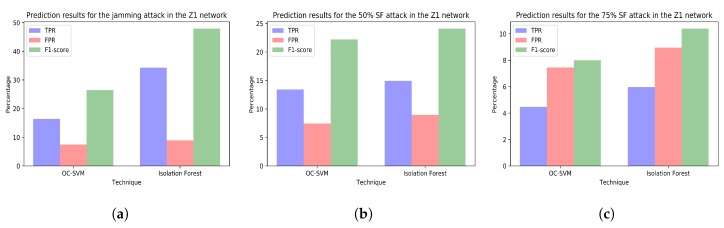
(**a**) prediction results for the jamming attack period using the variables from Z1 network (training with dataset *tr_z1* and test with dataset *te_z1_jam*); (**b**) prediction results for the 50% selective forwarding attack period using the variables from Z1 network (training with dataset *tr_z1* and test with dataset *te_z1_sf50*); (**c**) prediction results for the 75% selective forwarding attack period using the variables from Z1 network (training with dataset *tr_z1* and test with dataset *te_z1_sf75*).

**Figure 11:**
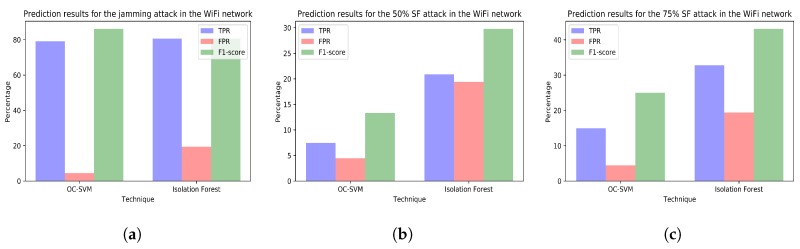
(**a**) prediction results for the jamming attack period using the variables from WiFi network (training with dataset *tr_rpi* and test with dataset *te_rpi_jam*); (**b**) prediction results for the 50% selective forwarding attack period using the variables from WiFi network (training with dataset *tr_rpi* and test with dataset *te_rpi_sf50*); (**c**) prediction results for the 75% selective forwarding attack period using the variables from WiFi network (training with dataset *tr_rpi* and test with dataset *te_rpi_sf75*).

**Figure 12:**
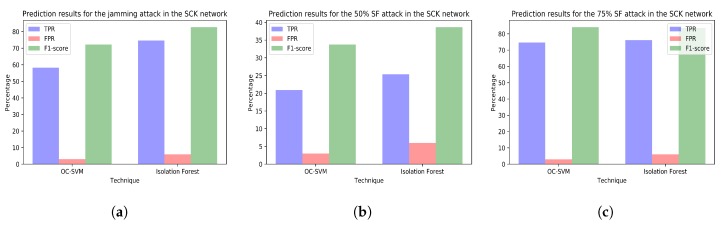
(**a**) prediction results for the jamming attack period using the variables from the SCK network (training with dataset *tr_sck* and test with dataset *te_sck_jam*); (**b**) prediction results for the 50% selective forwarding attack period using the variables from the SCK network (training with dataset *tr_sck* and test with dataset *te_sck_sf50*); (**c**) prediction results for the 75% selective forwarding attack period using the variables from the SCK network (training with dataset *tr_sck* and test with dataset *te_sck_sf75*).

**Figure 13:**
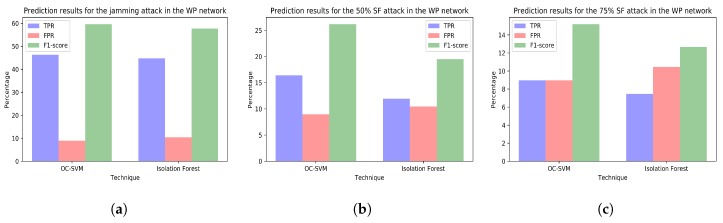
(**a**) prediction results for the jamming attack period using the variables from the WP network (training with dataset *tr_wp* and test with dataset *te_wp_jam*); (**b**) prediction results for the 50% selective forwarding attack period using the variables from the WP network (training with dataset *tr_wp* and test with dataset *te_wp_sf50*); (**c**) prediction results for the 75% selective forwarding attack period using the variables from the WP network (training with dataset *tr_wp* and test with dataset *te_wp_sf75*).

The Figure 10, Figure 11, Figure 12 and Figure 13 plot the prediction results for the three attacks comparing the two techniques creating a different model for each network.

## 6. Discussion

In this section, we discuss several issues related to the simulation and results presented above, presenting the difficulties encountered in the application of two different machine learning techniques in our simulated smart city environment.

**Using a single model versus several ones.** On the one hand, it can be seen that using a single model including variables from all the networks (i.e., models trained with dataset *tr_all*) usually results in good detection rates (Figure 9a,c). Moreover, dealing with a single model instead of dealing with multiple ones from different networks simplifies administrators’ task. In this way, administrators only have to review the prediction capabilities of a single model, retrain it if its performance decreases, and monitor the attack predictions of only one model that can be viewed as a summary of the anomalies occurring on a group of networks.

On the other hand, generating several models including different variables selected according to predefined meaningful reasons can help administrators to identify the causes of the anomalies or the affected equipment. For example, in the case of our jamming attack, conclusions derived from using all variables from all networks (Figure 9a) are limited to state that there is some malfunction affecting some element in the whole simulation environment. Nevertheless, studying the jamming attack predictions using a different model for each network (Figure 10a, Figure 11a, Figure 12a and Figure 13a) shows that conclusions drawn can be wider. For instance, the data analyst could doubtlessly see that there is an anomaly affecting the RPI and the SCK networks (Figure 11a and Figure 12a). Thus, administrators could easily identify the affected equipment.

Therefore, the difficulty here lies in deciding which approach is more appropriate for each smart city context, since there is a trade-off between simplifying the anomaly detection tasks and being able to derive more information from the results. Smart city administrators must be aware that there is no black box technique that can detect any kind of attack and the affected equipment in a scenario such as the smart city. In fact, a complex procedure and a framework are proposed in [25] in order to assist smart city administrators to select the most probable attack, and delimit corrupted data and affected devices in case of incident.

**Choosing the right machine learning technique.** If we performed a literature review on machine learning techniques for anomaly detection, we would probably come to the conclusion that SVM-based techniques are the ones that perform best. In our work, we have seen that both OC-SVM and isolation forest are suitable to detect the random jamming attack (Figure 9a) and OC-SVM models perform slightly better at detecting attacks in most of the situations (higher TPR). Moreover, in general, OC-SVM performs better at not misclassifying attack-free samples (lower FPR). This fact is important in smart cities so as to prevent overwhelming data analysts with too many false positives. However, in certain cases (Figure 10a and Figure 12a,b), the isolation forest models are capable of detecting more anomalies without producing more false positives. Moreover, the initialization of the isolation forest was simple and, unlike OC-SVM models, it did not require a grid search to find the best parameters.

Therefore, choosing one machine learning technique or another depends on the specific goals and characteristics of the environment. Data analysts should have tools to allow them to deploy different machine learning techniques in a simplified manner, so that different results can be compared and they can take advantage of several techniques simultaneously, each with its particular strengths.

**Attacks or malfunctions that sometimes go unnoticed.** None of the anomaly detection techniques is able to properly detect the selective forwarding attacks in the Z1 interface at the gateway. The study of the selective forwarding periods with the models including only variables from the Z1 network (Figure 10b,c) show poor detection results (i.e., low TPR or high FPR). It is worth noting that, although the model trained with dataset *tr_all* shows good detection results during the 75% selective forwarding attack period (Figure 9c), the malfunction raising signals of anomaly is the collapse of the SCK network and not the selective forwarding. This can also be seen examining the SCK network alone (Figure 12c). This enhances our previous conclusion that it is necessary not only to consider large models including all the possible variables, but also smaller ones including only meaningful variables.

Furthermore, after analyzing in depth the results and reviewing the normal operation of the Z1 network, we can conclude that the lack of good results detecting the selective forwarding attacks is due to the unreliable communication between the Z1 nodes. Figure 10a shows that even the jamming attack, which has been clearly detected in other networks, cannot be detected in the Z1 network. The most plausible cause for this is a large packet loss during the attack-free period. Additionally, it should be noted that, in this scenario, smart city administrators cannot notice the original packet loss, since the Z1 nodes have mechanisms to resend lost packets and, eventually, most of the information arrives at the gateway. From this fact, we can draw some conclusions. Firstly, city administrators should not trust that WSN providers properly configure their networks, even though sensor readings are apparently being received correctly at the city datacenters. Malfunctions, such as the one in the Z1 network, are detrimental for the nodes’ battery life and also for their already restringed processing power. In addition, these malfunctions fill the electromagnetic spectrum with useless packets, causing interference to other networks. Secondly, analysts should take into account that the response to the same attack can be different depending on the affected network. For instance, in these experiments, all the models should have detected the jamming attacks in all the networks, since the attacks aim at the 2.4 GHz band. However, the anomaly was clearly detected in just two of the networks.

**Availability of anomaly-free data.** Machine learning techniques use training processes to learn the mathematical models that are later used to classify data (normal samples or anomalies in our case). This training process, as previously shown, is based on the availability of a sufficiently large set of anomaly-free data. However, many anomaly detection strategies are designed and implemented once the networks have already been deployed. At this point, logically, it is difficult to guarantee that any set of real data is free of anomalies. Therefore, IT administrators and WSN providers should establish initial periods where networks are monitored carefully until they work as expected and networks generate anomaly-free data. Afterwards, given the fact that the behaviour of these networks evolves, models must be re-trained. At this moment, data analysts should bear in mind that networks can go through long periods of malfunctioning, so this also must be taken into account when re-training the models.

Additionally, because of the infeasibility of gathering a complete dataset of anomaly-free samples even in the most favorable circumstances, it is important that the detection techniques chosen to discover anomalies are capable to build the models from datasets which may include a certain amount of outliers (or abnormal samples), as is the case of the techniques used in this paper.

The challenge here lies in being able to obtain data that can be considered anomaly-free with a high degree of certainty when the smart city’s WSNs are already operating. New techniques or methodologies should be devised to solve this because otherwise there is no way that one can use any anomaly detection technique based on machine learning in this context.

**Aggregation and definition of time windows.** As we have seen in Figure 7, attacks have obvious effects when aggregating some variables in 4-h window intervals. Nonetheless, although this is practical to visually detect the attacks, it becomes a problem when training models. Using large time periods to aggregate data implies reducing the amount of samples that can be included in the datasets. Hence, this might become a problem due to a lack of samples to train, validate and test the models. In order to mitigate this problem, in addition to reducing the aggregation time intervals, analysts must consider using sliding windows (instead of fixed-interval windows as we did in our work), and also using mechanisms such as cross validation, as explained in Section 5.2.3, instead of creating different datasets for training and validation as it is being done in other scenarios with more available data.

## 7. Conclusions

Anomaly analysis in highly heterogeneous and dynamic environments, such as the smart city, requires sophisticated mechanisms. Research carried out to date has shown that machine learning-based techniques perform very well in these type of scenarios. However, in this paper, we have shown that putting this into practice is not easy at all.

There exists no black-box solution based on a multipurpose detection algorithm that is capable of performing a good anomaly detection regardless of the specific smart city environment. Quite the opposite, data analysts will face many difficulties and challenges, among which we have highlighted the following in this paper: choosing the right machine learning technique, analysing all variables through a single model or several of them, tackling with attacks or malfunctions that go unnoticed, obtaining anomaly-free data and defining the right aggregation time window.

In general, our work brings to light that analysts must be highly skilled both in data analysis and network administration and security because they must know how networks are supposed to behave. Therefore, multidisciplinary teams are necessary. Dealing with this from the very beginning, additionally, is of utmost importance (even though this is rarely the case) because otherwise it is difficult to obtain anomaly-free data and, without it, modelling the normal data behaviour is not possible.

Additionally, our work also reveals that there is still much work to do in the area of developing tools that promote and simplify the use of different types of anomaly detection techniques in real scenarios.

Finally, in our work, we have used two specific well-known machine learning algorithms, but it is worth noting that the encountered difficulties mentioned in our discussion would have been the same using other techniques. The stated difficulties or challenges are not motivated by any particular algorithm feature but instead to the fundamental way machine learning algorithms work.

## Figures and Tables

**Table 1:** Details of the testbed.

**Zolertia Network**
• Device models: Z1 [47] • Network topology: mesh • Transmit power: Z1-0 and Z1-7 at 0 dBm; Z1-1 to Z1-6 at −15 dBm • Communication protocol: 6LoWPAN • Frequency and channel: 2.4 GHz, channel 15
**Libelium network**
• Device models: Waspmote [45] • Network topology: star • Transmit power: 17 dBm • Communication protocol: ZigBee • Frequency and channel: 2.4 GHz, channel 14
**RPI network**
• Device models: Raspberry Pi 3 [48] • Network topology: star • Transmit power: 15 dBm • Communication protocol: WiFi • Frequency and channel: 2.4 GHz, channel 1
**Smart citizen kit (SCK)**
• Device models: SCK 1.1 [49] • Network topology: star • Transmit power: 15 dBm • Communication protocol: WiFi • Frequency and channel: 2.4 GHz, channel 1
**Data warehouse**
• Splunk 6.5.1

**Table 2:** Summary of the variables resulting from our aggregation. These variables are the result of aggregating data from all nodes in each network in 30 min intervals.

**Zolertia Network**
• Amount of lost packets • Amount of received packets • Average time between consecutive packets • Number of times that the channel was occupied before transmitting
**Libelium network**
• Amount of lost packets • Amount of received packets • Average time between consecutive packets • Average percentage of battery consumption
**RPI network**
• Amount of lost packets • Amount of received packets • Average time between consecutive packets • Signal level
**Smart citizen kit (SCK)**
• Amount of lost packets • Amount of received packets • Average time between consecutive packets

**Table 3:** Metrics summary.

**True Positive Rate (TPR)**	Also called detection rate or sensitivity, measures the percentage of properly detected attacks	TP/(TP + FN)
**False Positive Rate (FPR)**	Also called fall-out, measures the percentage of misclassified normal samples	FP/(FP + TN)
**F1-score**	Measures the number of true positives over the average of predicted and real positives	2TP/(2TP + FP + FN)
